# Acculturation and Plasma Fatty Acid Concentrations in Hispanic and Chinese-American Adults: The Multi-Ethnic Study of Atherosclerosis

**DOI:** 10.1371/journal.pone.0149267

**Published:** 2016-02-12

**Authors:** Cassandra S. Diep, Rozenn N. Lemaitre, Tzu-An Chen, Tom Baranowski, Pamela L. Lutsey, Ani W. Manichaikul, Stephen S. Rich, David E. St-Jules, Brian T. Steffen, Michael Y. Tsai, David S. Siscovick, Alexis C. Frazier-Wood

**Affiliations:** 1 USDA/ARS Children’s Nutrition Research Center, Department of Pediatrics, Baylor College of Medicine, Houston, TX, United States of America; 2 Department of Health Disparities Research, The University of Texas MD Anderson Cancer Center, Houston, TX, United States of America; 3 Cardiovascular Health Research Unit, Department of Medicine, University of Washington, Seattle, WA, United States of America; 4 Division of Epidemiology & Community Health, School of Public Health, University of Minnesota, Minneapolis MN, United States of America; 5 Center for Public Health Genomics, University of Virginia, Charlottesville, VA, United States of America; 6 Department of Nutrition, Harvard School of Public Health, Boston, MA, United States of America; 7 Department of Laboratory Medicine and Pathology, University of Minnesota, Minneapolis, MN, United States of America; 8 New York Academy of Medicine, New York, NY, United States of America; University of Milan, ITALY

## Abstract

**Background:**

Acculturation to the U.S. is associated with increased risk of cardiovascular disease, but the etiologic pathways are not fully understood. Plasma fatty acid levels exhibit ethnic differences and are emerging as biomarkers and predictors of cardiovascular disease risk. Thus, plasma fatty acids may represent one pathway underlying the association between acculturation and cardiovascular disease. We investigated the cross-sectional relationship between acculturation and plasma phospholipid fatty acids in a diverse sample of Hispanic- and Chinese-American adults.

**Methods and Findings:**

Participants included 377 Mexican, 320 non-Mexican Hispanic, and 712 Chinese adults from the Multi-Ethnic Study of Atherosclerosis, who had full plasma phospholipid assays and acculturation information. Acculturation was determined from three proxy measures: nativity, language spoken at home, and years in the U.S., with possible scores ranging from 0 (least acculturated) to 5 (most acculturated) points. α-Linolenic acid, linoleic acid, eicosapentaenoic acid, docosahexaenoic acid, and arachidonic acid were measured in fasting plasma. Linear regression models were conducted in race/ethnicity-stratified analyses, with acculturation as the predictor and plasma phospholipid fatty acids as the outcome variables. We ran secondary analyses to examine associations between acculturation and dietary fatty acids for comparison. Covariates included age, gender, education, and income. Contrary to our hypothesis, no statistically significant associations were detected between acculturation and plasma phospholipid fatty acids for Chinese, non-Mexican Hispanic, or Mexican participants. However, acculturation was related to dietary total n-6 fatty acids and dietary n-3/n-6 ratios in expected directions for Mexican, non-Mexican Hispanic, and combined Hispanic participants. In Chinese individuals, acculturation was unexpectedly associated with lower arachidonic acid intake.

**Conclusion:**

Absence of associations between acculturation and plasma phospholipid fatty acids suggests that changes in the plasma phospholipid fatty acids studied do not account for the observed associations of acculturation to the U.S. and cardiovascular disease risk. Similar findings were observed for eicosapentaenoic acid and docosahexaenoic acid, when using dietary intake. However, the observed associations between dietary n-6 fatty acids and acculturation in Hispanic individuals suggest that dietary intake may be more informative than phospholipids when investigating acculturation effects. In Chinese individuals, acculturation may have a possible protective effect through decreased arachidonic acid intake. Further research on dietary fatty acids and other cardiovascular disease biomarkers is needed to identify possible etiologic mechanisms between acculturation and cardiovascular disease.

## Introduction

Acculturation, or the process by which foreign-born individuals adopt the culture and behaviors of a new environment, has been associated with increased cardiovascular disease (CVD) incidence and risk factors in the U.S. [1–7]. The pathways between acculturation and CVD risk remain largely unknown, and may include changes in regular diet or physical activity [8–15], increased smoking [16], nonadherence to antihypertensive medication [17], or acculturative stress [18]. Examining associations between acculturation and biomarkers of CVD risk may provide insight into which metabolic pathways are disturbed with acculturation and discover additional intervention targets for improving the health of acculturating populations.

Plasma phospholipid fatty acids (PLFAs), particularly polyunsaturated FAs such as omega-3 (n-3) and possibly omega-6 (n-6) FAs [19, 20], are recognized as modifiable biomarkers of CVD risk [21–25]. A controversial meta-analysis of 32 prospective cohort studies found eicosapentaenoic acid (EPA) (n-3 FA), docosahexaenoic acid (DHA) (n-3 FA), and arachidonic acid (AA) (n-6 FA) were associated with lower coronary heart disease risk [26]. Studies in *dietary* polyunsaturated FAs were less consistent [26], as there were statistically non-significant associations reported between dietary intake of n-3 and n-6 FAs and coronary heart disease, potentially due to measurement error in self-reported questionnaires used to assess FA consumption. With research showing associations between acculturation and increased CVD, as well as between plasma PLFAs and increased CVD, one possible avenue through which acculturation may increase CVD risk is through changes in FA profiles. However, little is known about the potential effect of acculturation on plasma PLFAs.

Asian and Hispanic/Latino descent populations are the fastest-growing immigrant groups in the U.S. [27, 28], who have experienced both acculturation and increased CVD risks. Asians grew by 2.9% from 2011 to 2012, constituting 5.8% of the total U.S. population in 2012; Hispanics/Latinos grew by 2.2% and constituted 16.9% of the total U.S. population in 2012 [27, 28]. Furthermore, Hispanic/Latino or Asian subgroups have different background lifestyles, acculturation experiences, and health outcomes [29].

In this study, we investigated the association of acculturation and plasma PLFA concentrations in a sample of Mexican, non-Mexican Hispanic, and Chinese-American (one of the largest and quickly-growing Asian subgroups in the U.S.) adults from the Multi-Ethnic Study of Atherosclerosis (MESA). A characteristic of westernized diets is a high ratio of n-6 FAs to n-3 FAs [1, 30]. For this reason, we hypothesized that acculturation levels would be positively associated with fasting plasma phospholipid n-6 FAs and inversely associated with fasting plasma phospholipid n-3 FAs. In secondary analyses, we also examined whether associations between acculturation and plasma PLFAs were mirrored by associations between acculturation and dietary FA intake.

## Materials and Methods

### Study population

We used cross-sectional data from MESA, a prospective cohort study of subclinical CVD among four ethnic populations [31]. Started in 2000, MESA includes 6,814 participants aged 45–84 years, who were free of clinical CVD at baseline, from six areas in the U.S.: New York, New York; Baltimore, Maryland; Chicago, Illinois; Los Angeles, California; St. Paul, Minnesota; and Forsyth County, North Carolina. Study procedures were approved by the Institutional Review Boards of all field centers (i.e., Columbia University; Johns Hopkins University; Northwestern University; University of California, Los Angeles; University of Minnesota; and Wake Forest University) and the Coordinating Center (University of Washington), and all participants provided written informed consent. All study analyses were conducted on de-identified data.

The MESA sample in the current analyses contains data from the baseline/exam 1 visit when acculturation was measured. Our analytic sample included only participants from the subset with FA data; plasma PLFAs were measured at baseline in a randomly selected subset of 2,856 participants. We further excluded those without full acculturation and diet data and participants who did not self-identify as Mexican, non-Mexican Hispanic (*e*.*g*., Cubans, Puerto Ricans, Dominicans), and/or Chinese. The final sample size was 1,409 self-identified participants: 377 Mexican, 320 non-Mexican Hispanic, and 712 Chinese.

### Measures

#### Demographics

Demographic characteristics, such as age, sex, race/ethnicity, education, and income, were collected by self-report using questionnaires. Questionnaires, available in English, were also translated to Spanish and Chinese by certified translators and reviewed by bilingual study investigators, staff, and a multicultural research office. Race/ethnicity was self-reported using the same race and ethnicity questions as in the 2000 U.S. Census. Participants who selected Hispanic were asked to choose their specific group (*e*.*g*., Mexican, Dominican, Puerto Rican).

#### Acculturation

As previously done [32], an acculturation score was constructed from three proxy measures: nativity, language spoken at home, and years in the U.S. Nativity and years in the U.S. were combined and scored as U.S.-born (3 points), foreign-born and lived in the U.S. at least 20 years (2 points), foreign-born and lived in the U.S. 10–19 years (1 point), or foreign-born and lived in the U.S. less than 10 years (0 points). A separate score was given for language spoken at home: English (2 points), English and Chinese/Spanish (1 point), or non-English language (0 points). The two scores were summed to obtain an acculturation score from 0 (least acculturated) to 5 (most acculturated).

#### Plasma Phospholipid Fatty Acids

Plasma PLFAs were measured in plasma phospholipids; the ones of interest for this study were α-linolenic acid (ALA), linoleic acid (LA), EPA, DHA, and AA. These FAs were measured in EDTA plasma frozen at -70˚C using samples collected after a 12-hour fast [33, 34]. Plasma phospholipids were isolated by thin layer chromatography, with FAs being subsequently separated by gas chromatography. The Collaborative Studies Clinical Laboratory at Fairview-University Medical Center (Minneapolis, MN) performed the FA assays; details of sample shipping, repository, processing, and extraction are published elsewhere [31, 33, 35]. Individual FAs were expressed as a percentage of total FAs.

#### Dietary Fatty Acids

Dietary FAs were assessed from a self-administered food frequency questionnaire (FFQ). The FFQ was a modified version of the Insulin Resistance Atherosclerosis Study FFQ, which was previously validated in non-Hispanic whites, Hispanics, and African Americans [36, 37], and included additional items to capture the dietary intake of Chinese Americans. For each food item, individuals indicated the average serving size and frequency of consumption. Total dietary intake of FAs was calculated using weighted recipes from the Nutrition Data System for Research (NDSR, University of Minnesota, Minneapolis, MN) and estimated per 100 g of food [38]. Those values were then multiplied by individual intake frequency and age-, sex-, and portion size-specific gram weights for each food. Reported intakes of dietary FAs were analyzed as absolute amounts (mg/d) for FAs that are episodically-consumed (EPA, DHA, EPA+DHA), and in relation to energy intake (per 1,000 kcal) for all others [39]. In addition, n-3 FAs were analyzed in relation to n-6 FA intake.

#### Anthropometrics and other health outcomes

Height was measured to the nearest 0.1 cm and weight to 0.5 kg by trained personnel and converted into body mass index (BMI) using the formula: weight (kg) / height^2^ (m^2^). Waist circumference was measured to the nearest 0.1 cm. Serum glucose was measured by a Vitros analyzer (Johnson & Johnson Clinical Diagnostics Inc., Rochester, NY). Serum insulin was measured by a radioimmunoassay method using the Linco Human Insulin-Specific RIA Kit (Linco Research Inc., St. Charles, MO). The homeostasis model of insulin resistance (HOMA-IR) was calculated as: insulin (mU/l) x (glucose [mg/dl] x 0.055)/22.5 [40].

#### Physical activity

Physical activity was assessed using a questionnaire adapted from the Cross-Cultural Activity Participation Study [31]. For this study, physical activity was defined as a total of the metabolic equivalent (MET) hours per day reported from leisure and occupational activities.

#### Statistical analyses

Plasma PLFA variables, with the exception of total n-6, DHA, and LA, were non-normally distributed, as well as insulin, glucose, and HOMA-IR. These variables were log transformed for the analyses. Values that were three standard deviations from the mean for glucose were excluded prior to analyses.

We performed linear regressions to assess the association of acculturation with individual plasma PLFAs in separate models. We also ran secondary analyses on dietary FAs by ethnicity and acculturation for comparison. In all models, the predictor was the composite acculturation score. In model 1, age, sex, highest educational level, and total gross family income were included as covariates. Model 2 included the model 1 predictors and covariates, plus fasting insulin levels, BMI, waist circumference, and physical activity. Study site was not included as a covariate, as this was strongly correlated with ethnicity and acculturation. Multicollinearity tests were performed to determine if any of the covariates and predictor variables were highly correlated. Based on variance inflation factors, there was no problem with collinearity in the data.

All statistical analyses were performed using Statistical Analysis System (SAS version 9.4, SAS Institute Inc., Cary, NC, 2014), conducted separately for Mexican Hispanic, non-Mexican Hispanic, and Chinese participants. Analyses for all Hispanics (Mexican and non-Mexican) were performed, while controlling for Mexican background. Statistical significance was based upon a 5% false discovery rate (FDR) and presented as corrected q-values [41].

## Results

The total sample comprised 1,409 MESA participants of Mexican (*n* = 377), non-Mexican Hispanic (*n* = 320), or Chinese (*n* = 712) descent (Table 1). The mean age was approximately 62 years, and there was nearly equal representation from males and females. Overall, based on time in the U.S., primary language spoken at home, and acculturation score, those of Mexican descent were more acculturated than non-Mexican Hispanic and Chinese participants (*P*<0.001 for all three variables). In addition, there were statistically significant differences in PLFAs and dietary FAs between ethnic groups (*P*<0.05).

**Table 1:** Selected demographic and health characteristics of MESA sample: 2000–2002 (*n* = 1409).

	Mexican (*n* = 377)	Non-Mexican Hispanic (*n* = 320)	Chinese (*n* = 712)
Age (y)	61.5 ± 10.2^a^	61.2 ± 10.4	62.4 ± 10.4
Gender (% male)	50.6	45.7	49.2
Time in the U.S. (%)^b^			
Born in the U.S.	50.4	7.8	3.6
At least 20 years	13.8	17.2	24.8
10–19 years	5.2	12.3	30.5
<10 years	30.7	62.7	41.2
Primary language at home (%)^b^			
English only	40.9	15.1	5.6
English + another	16.9	14.9	7.1
Non English only	42.2	70.0	87.3
Acculturation score (%)^b^			
5 (most acculturated)	36.9	4.8	2.5
4	14.0	10.6	3.2
3	7.7	11.2	5.8
2	23.6	46.4	34.9
1	5.8	13.4	29.6
0 (least acculturated)	12.0	13.7	24.0
BMI (kg/m^2^)^b^	29.9 ± 5.2	28.9 ± 5.0	24.0 ± 3.3
Insulin (mU/L)^c^	12.3 ± 12.8	11.3 ± 18.6	9.6 ± 12.5
Waist circumference (cm)^b^	101.9 ± 13.0	99.2 ± 13.1	87.2 ± 9.8
Circulating fatty acids (% of total phospholipid fatty acids)			
EPA+DHA^b^	3.4 ± 1.1	4.8 ± 1.7	6.4 ± 2.5
EPA^b^	0.6 ± 0.3	0.8 ± 0.6	1.3 ± 1.3
DHA^b^	2.8 ± 0.9	4.0 ± 1.3	5.2 ± 1.5
ALA^d^^,^^e^	0.2 ± 0.1	0.2 ± 0.1	0.2 ± 0.1
LA^d^^,^^e^	23.0 ± 2.9	20.7 ± 3.2	23.4 ± 3.6
AA^b^	11.0 ± 2.3	12.4 ± 2.7	10.5 ± 2.2
Dietary fatty acids (mg/day)			
EPA+DHA^c^^,^^e^	89.3 ± 91.0	104.0 ± 103.7	133.6 ± 106.7
EPA^b^	27.9 ± 36.2	36.4 ± 41.7	51.3 ± 46.3
DHA^c^^,^^e^	61.4 ± 56.2	67.5 ± 63.4	82.2 ± 61.9
ALA^c^^,^^e^^,^^f^	1040.8 ± 561.4	913.2 ± 609.1	641.7 ± 418.8
LA^c^^,^^d^	9741.0 ± 6418.3	7298.5 ± 5855.7	7239.1 ± 4256.3
AA^d^^,^^e^^,^^g^	124.6 ± 84.2	98.5 ± 80.9	105.3 ± 67.0

AA, arachidonic acid; ALA, α-linolenic acid; BMI, body mass index; DHA, docosahexaenoic acid; EPA, eicosapentaenoic acid; LA, linoleic acid; MESA, Multi-Ethnic Study of Atherosclerosis.

^a^ Mean ± SD (all such values).

^b^ Significant differences for all three pairwise comparisons at *P*<0.001.

^c^ Significant differences between Mexican and Chinese at *P*<0.001.

^d^ Significant differences between Mexican and non-Mexican Hispanic at *P*<0.001.

^e^ Significant differences between non-Mexican Hispanic and Chinese at *P*<0.001.

^f^ Significant differences between Mexican and non-Mexican Hispanic at *P*<0.05.

^g^ Significant differences between Mexican and Chinese at *P*<0.05.

Acculturation was not significantly associated with any plasma PLFAs in model 1, regardless of racial/ethnic subgroup (all *q*≥0.432; Table 2). We observed similar results with model 2 and when combining all Hispanics. We also checked whether the regression coefficients differed across subgroup by testing the interaction effects. When controlling for covariates, there was a significant difference in regression coefficients between non-Mexican Hispanic and Chinese participants (*P*<0.01).

**Table 2:** Association between acculturation and plasma polyunsaturated fatty acids among MESA participants: 2000–2002 (*n* = 1409)^a^.

	Mexican (*n* = 377)				Non-Mexican Hispanic (*n* = 320)				Chinese (*n* = 712)			
Variable	*β*^b^	SE	*P*-value	*q*-value	*β*^b^	SE	*P*-value	*q*-value	*β*^b^	SE	*P*-value	*q*-value
Plasma fatty acids												
n-3 fatty acids	-0.102	0.011	0.126	0.432	-0.035	0.014	0.539	0.703	-0.056	0.012	0.160	0.432
n-6 fatty acids	0.107	0.095	0.108	0.432	0.063	0.130	0.267	0.515	-0.070	0.112	0.077	0.432
n-3/n-6	-0.121	0.012	0.069	0.432	-0.048	0.016	0.402	0.678	-0.029	0.014	0.464	0.703
EPA+DHA	-0.109	0.012	0.103	0.432	-0.034	0.015	0.547	0.703	-0.056	0.012	0.156	0.432
EPA	-0.014	0.018	0.835	0.889	0.010	0.027	0.856	0.889	-0.013	0.025	0.744	0.837
DHA	-0.074	0.037	0.267	0.515	-0.021	0.057	0.709	0.832	-0.060	0.050	0.128	0.432
ALA	0.078	0.013	0.236	0.515	0.036	0.019	0.530	0.703	-0.029	0.013	0.474	0.703
LA	0.004	0.113	0.950	0.950	-0.029	0.144	0.614	0.754	-0.035	0.121	0.379	0.678
AA^c^	0.117	0.091	0.083	0.432	0.102	0.126	0.085	0.432	-0.047	0.075	0.238	0.515

AA, arachidonic acid; ALA, α-linolenic acid; BMI, body mass index; DHA, docosahexaenoic acid; EPA, eicosapentaenoic acid; HOMA-IR, homeostasis model of insulin resistance; LA, linoleic acid; MESA, Multi-Ethnic Study of Atherosclerosis.

^a^ Covariates included age and gender.

^b^ Standardized beta coefficients are shown.

^c^ Significant differences between non-Mexican Hispanic and Chinese at *P*<0.01.

We ran secondary analyses of dietary FAs for comparison: acculturation was significantly associated with increased n-6 FA intake, decreased n-3/n-6 FA intake, and increased LA intake in Mexican and non-Mexican Hispanic participants, as well as decreased AA intake in Chinese participants in model 1 (all *q*<0.05; Table 3). The results were similar in model 2. When combining all Hispanics, the results were slightly different than those observed for Mexican and non-Mexican Hispanic participants. Along with increased n-6 FA (*β* = 0.178, *q*<0.001), decreased n-3/n-6 FA (*β* = -0.125, *q*<0.001), and increased LA (*β* = 0.177, *q*<0.001) intake, acculturation was significantly associated with increased AA (*β* = 0.101, *q* = 0.005) intake among combined Hispanics in model 1, with similar results in model 2. The dietary measures and plasma PLFAs correlated well for DHA (*ρ* = 0.30, *P*<0.001), EPA (*ρ* = 0.35, *P*<0.001), and EPA+DHA (*ρ* = 0.35, *P*<0.001), while the other correlations were either small (0.05 for ALA, 0.09 for total n-3, 0.17 for total n-6, and 0.21 for LA; all *P*<0.05) or not statistically significant (*P*>0.05 for n-3/n-6 and AA), which suggests that only dietary EPA, DHA, and EPA+DHA had reasonable correlations with their biomarkers in our dataset.

**Table 3:** Association between acculturation and dietary polyunsaturated fatty acids among MESA participants: 2000–2002 (*n* = 1409)^a^.

	Mexican (*n* = 377)				Non-Mexican Hispanic (*n* = 320)				Chinese (*n* = 712)			
Variable	*β*^b^	SE	*P*-value	*q*-value	*β*^b^	SE	*P*-value	*q*-value	*β*^b^	SE	*P*-value	*q*-value
Dietary fatty acids												
n-3 fatty acids	0.054	0.007	0.221	0.497	0.013	0.011	0.763	0.858	0.063	0.010	0.101	0.248
n-6 fatty acids^c^^,^^d^	0.197	0.007	<0.001	<0.001	0.132	0.011	0.002	0.014	0.040	0.009	0.299	0.505
n-3/n-6	-0.152	0.007	0.001	0.009	-0.119	0.011	0.006	0.023	0.025	0.010	0.515	0.695
EPA+DHA	0.009	0.024	0.844	0.912	0.019	0.035	0.673	0.790	0.032	0.030	0.415	0.659
EPA	-0.004	0.034	0.933	0.933	-0.025	0.042	0.577	0.742	0.044	0.039	0.265	0.505
DHA	0.020	0.023	0.664	0.790	0.032	0.033	0.468	0.665	0.030	0.028	0.445	0.665
ALA^d^	0.047	0.007	0.284	0.504	0.004	0.011	0.922	0.933	0.086	0.011	0.023	0.078
LA^c^^,^^d^	0.196	0.007	<0.001	<0.001	0.130	0.011	0.003	0.016	0.043	0.010	0.257	0.505
AA	0.089	0.014	0.051	0.138	0.094	0.022	0.035	0.105	-0.111	0.018	0.004	0.018

AA, arachidonic acid; ALA, α-linolenic acid; BMI, body mass index; DHA, docosahexaenoic acid; EPA, eicosapentaenoic acid; HOMA-IR, homeostasis model of insulin resistance; LA, linoleic acid; MESA, Multi-Ethnic Study of Atherosclerosis.

^a^ Covariates included age and gender.

^b^ Standardized beta coefficients are shown.

^c^ Significant differences between Mexican and Chinese at *P*<0.01.

^d^ Significant differences between non-Mexican Hispanic and Chinese at *P*<0.05.

Based on a sensitivity power analysis using G*Power (version 3.1.9.2, Heinrich*-*Heine*-*University, Düsseldorf, Germany, 2014), assuming a conservative (Bonferroni-corrected) alpha = 0.005 and the smallest sample size of 320 (non-Mexican Hispanics), we had 80% power to detect a small effect size of *f*^*2*^ *=* 0.042. This implies that if there was a true relationship between acculturation and the FAs that we were not powered to detect, acculturation accounted for less than 4% of the FA variances and so may not be clinically meaningful.

Scatter plots for each plasma PLFA against acculturation score did not reveal any differences between the acculturation groups. In addition, the acculturation score was positively associated with BMI, which theoretically and empirically should be related [42–45], in our samples of Chinese (standardized *β* = 0.117, *P* = 0.001), Mexican (standardized *β* = 0.103, *P* = 0.003), and non-Mexican Hispanic (standardized *β* = 0.091, *P* = 0.020) participants, providing validity to this acculturation measure.

## Discussion

To our knowledge, the current study was the first to test associations between acculturation and plasma PLFAs as a possible pathway between acculturation and CVD, in a sample of Chinese and Hispanic MESA participants. No significant relationships were detected between acculturation and plasma phospholipid polyunsaturated FAs. These findings suggest that changes in plasma polyunsaturated FAs are not a pathway through which acculturation to the U.S. may modulate CVD risk.

Associations between acculturation and polyunsaturated FA intake have been suggested from ecological studies. For example, humans used to consume a diet with an approximate 1:1 ratio of n-6 to n-3 FAs [46, 47], but recently, westernized diets have been characterized by a ratio of between 10:1 and 20:1 [46–48]. With this increase in ratio, the human physiological state becomes more proinflammatory, thus theoretically increasing the risks of CVD and other diseases characterized by inflammation [30, 49] and illustrating a potential pathway through which acculturation may influence CVD risk. These associations between acculturation and a westernized diet were not supported by plasma PLFA levels in Chinese, Mexican, non-Mexican Hispanic, or combined Hispanic individuals in this study; however, they appeared supported by associations of *dietary* total n-6 polyunsaturated FA levels with higher acculturation, and dietary n-3/n-6 ratios with lower acculturation, in Mexican, non-Mexican Hispanic, and combined Hispanic individuals. In Chinese individuals, acculturation was associated with lower AA intake, suggesting a possible protective effect through decreased AA intake. Plasma FAs reflect both dietary intake and metabolic processes; our results may suggest that the effects of acculturation on FA metabolism are minimal and that the focus should be on dietary intake, at least for some FAs. Correlations between dietary and plasma FAs showed that dietary total n-6 polyunsaturated FAs and dietary AA in this study did not correlate well with their biomarker, questioning the comparability of the diet and plasma phospholipid measurements for these FAs. On the other hand, EPA, DHA, and EPA+DHA showed modest correlations [50], and consistent null findings with both diet and biomarkers suggest that acculturation is not associated with very long chain n-3 polyunsaturated FAs for Mexican, non-Mexican Hispanic, combined Hispanic, or Chinese individuals. Research is needed to further investigate the relationship between acculturation and other dietary FAs.

Limitations of the current study include first, the cross-sectional design. Longitudinal research is needed to assess the associations of acculturation to FAs and CVD, as relationships may manifest over time. Second, the acculturation score was based on nativity, language spoken at home, and years in the U.S., and these three variables may not adequately capture acculturation. However, we found positive associations between our acculturation measure and weight status, which is consistent with other studies on acculturation [42–45]. Relatedly, we assumed linearity in the acculturation score, for which use of a single unit may have yielded misleading findings (e.g., if there was no change in a particular FA until a score of 4). We created scatter plots for each FA against acculturation score and found no differences between any of the acculturation groups; thus, the way we classified acculturation was not misleading. In addition, although one of the strengths of this paper is focusing on plasma PLFAs instead of self-reported dietary FAs, there may have been measurement error or limited variability in some of the plasma PLFAs in these populations, which may have contributed to our null findings. Next, findings from this study cannot be generalized to other racial/ethnic populations in the U.S. or around the world, who also undergo acculturation or westernization. Lastly, dietary FAs were measured using a FFQ. Dietary FA assessment with the FFQ includes measurement error, and dietary assessment validity may vary among ethnic groups.

The present study contributes to research on acculturation and CVD risk factors by being the first to investigate plasma phospholipid polyunsaturated FAs, in relation to acculturation, in Asian/Hispanic subgroups. Associations were not detected with very long chain n-3 FAs measured in plasma and diet, suggesting that acculturation may not be related to increased CVD risk through changes in EPA and DHA. Research is needed to investigate the association of acculturation with other CVD biomarkers of risk, such as lipids, lipoproteins, and oxidized FAs, as well as with n-6 dietary FAs.
